# A propofol binding site in the voltage sensor domain mediates inhibition of HCN1 channel activity

**DOI:** 10.1126/sciadv.adr7427

**Published:** 2025-01-03

**Authors:** Verena Burtscher, Lei Wang, John Cowgill, Zi-Wei Chen, Christopher Edge, Edward Smith, Yongchang Chang, Lucie Delemotte, Alex S. Evers, Baron Chanda

**Affiliations:** ^1^ Department of Anesthesiology, Washington University School of Medicine, St. Louis, MO 63110, USA.; ^2^ Center for Membrane Excitability Disorders, Washington University School of Medicine, St. Louis, MO 63110, USA.; ^3^ Department of Anesthesiology, Union Hospital, Tongji Medical College, Huazhong University of Science and Technology, Wuhan 430022, China.; ^4^ Key Laboratory of Anesthesiology and Resuscitation (Huazhong University of Science and Technology), Ministry of Education, Wuhan 430022, China.; ^5^ Department of Biochemistry and Biophysics, SciLifeLab, Stockholm University, 17121 Solna, Sweden.; ^6^ Department of Life Sciences, Imperial College, London SW7 2AZ, UK.; ^7^ Department of Biophysics, Imperial College of Science, Medicine and Technology, London SW7 2AZ, UK.; ^8^ Department of Applied Physics, SciLifeLab, KTH Royal Institute of Technology, 17121 Solna, Sweden.; ^9^ Department of Developmental Biology, Washington University School of Medicine, St. Louis, MO 63110, USA.; ^10^ Department of Neuroscience, Washington University School of Medicine, St. Louis, MO 63110, USA.; ^11^ Department of Biochemistry and Molecular Biophysics, Washington University School of Medicine, St. Louis, MO 63110, USA.

## Abstract

Hyperpolarization-activated and cyclic nucleotide–gated (HCN) ion channels are members of the cyclic nucleotide–binding family and are crucial for regulating cellular automaticity in many excitable cells. HCN channel activation contributes to pain perception, and propofol, a widely used anesthetic, acts as an analgesic by inhibiting the voltage-dependent activity of HCN channels. However, the molecular determinants of propofol action on HCN channels remain unknown. Here, we use a propofol-analog photoaffinity labeling reagent to identify propofol binding sites in the human HCN1 isoform. Mass spectrometry analyses combined with molecular dynamics simulations show that a binding pocket is formed by extracellularly facing residues in the S3 and S4 transmembrane segments in the resting voltage-sensor conformation. Mutations of residues within the putative binding pocket mitigate or eliminate voltage-dependent modulation of HCN1 currents by propofol. Together, these findings reveal a conformation-specific propofol binding site that underlies voltage-dependent inhibition of HCN currents and provides a framework for identifying highly specific modulators of HCN channel gating.

## Teaser

Propofol inhibits pain by binding to a specific site on HCN channels, offering insights for developing nonsedative treatments.

## INTRODUCTION

Hyperpolarization-activated and cyclic nucleotide–gated (HCN) ion channels are expressed in a range of excitable cells in the nervous system and heart (*1*–*3*). The transmembrane regions of these channels are structurally homologous to voltage-gated K^+^ channels, but two key functional characteristics enable them to play a unique role in cellular excitability (*4*). First, in contrast to other members of the voltage-gated ion channel superfamily, these channels open upon hyperpolarization rather than depolarization (*5*, *6*). Second, these channels are much less selective for K^+^ (*E*_rev_ = −30 mV) than a typical K^+^ channel. Since the electrochemical driving force is much larger for sodium than for potassium at hyperpolarized potentials, the opening of HCN channels results in inward sodium currents. These currents mediated by HCN channels are referred to as either *I*_f_ (in the heart) or *I*_h_ (neurons) currents, and are the primary contributors to the automaticity of action potentials in rhythmic circuits such as those found in the cardiac pacemaker cells (*5*–*9*). Increased activity of HCN channels in these circuits at hyperpolarized potentials drives slow membrane depolarization, bringing the cell membrane to the threshold for the next action potential.

In neurons, HCN channels regulate electrical excitability, and dysfunction of these channels due to mutations is associated with aberrant excitability and inherited epilepsies (*10*). During neuropathic pain, HCN channel activity increases because of up-regulation (*11*–*13*) and elevated adenosine 3′,5′-monophosphate (cAMP) levels resulting from inflammation (*14*). Blockers of HCN channels such as ZD-7288 can reverse neuropathic hypersensitivity in animal models without altering acute pain threshold. Propofol, an intravenous anesthetic, also inhibits *I*_h_ currents in hippocampal pyramidal neurons and cortical pyramidal neurons at clinically relevant concentrations (*15*, *16*). Propofol analogs, such as the alkylphenol 2,6-di-*tert*-butylphenol, impede the gating of HCN1 channels without affecting either γ-aminobutyric acid type A (GABA_A_) or glycine receptors; they are also antihyperalgesic in animal models of neuropathic pain (*17*), indicating a role for HCN channel inhibition in the analgesic effect of propofol. The HCN1 channel provides an attractive target for the development of an isoform-specific analgesic, given its expression in dorsal root ganglia and its absence in the sinoatrial node, where HCN2 and HCN4 mediate cardiac pacemaker activity (*17*, *18*).

Propofol inhibits HCN channel activity by shifting the voltage dependence of channel opening toward more hyperpolarized potentials (*19*–*21*). However, neither the nature of the propofol binding pocket nor the molecular mechanism of propofol action has been elucidated, hindering the development of analogs that are more specific modulators of HCN1 channel activity. In this study, we used a propofol-analog photoaffinity labeling (PAL) reagent combined with tandem mass spectrometry (MS/MS) to localize a propofol binding site in HCN1 channels to the S3-S4 segment of the voltage sensor domain (VSD). Atomistic molecular dynamics (MD) simulations on the closed-state structure of the HCN1 channel also show that propofol binds to a pocket shaped by the residues near the extracellular region of the S3-S4 segment. This binding pocket is notably absent in the activated voltage-sensor structure of the HCN channel. Mutagenesis studies combined with electrophysiological measurements highlight the critical role of multiple residues in this binding pocket. Together, these findings suggest that propofol binding stabilizes the S3-S4 helices in the resting-state conformation, thereby shifting the voltage dependence of HCN channel activation to more hyperpolarized potentials.

## RESULTS

### MS/MS analysis of AziPm-labeled sites

**Fig. 1.**
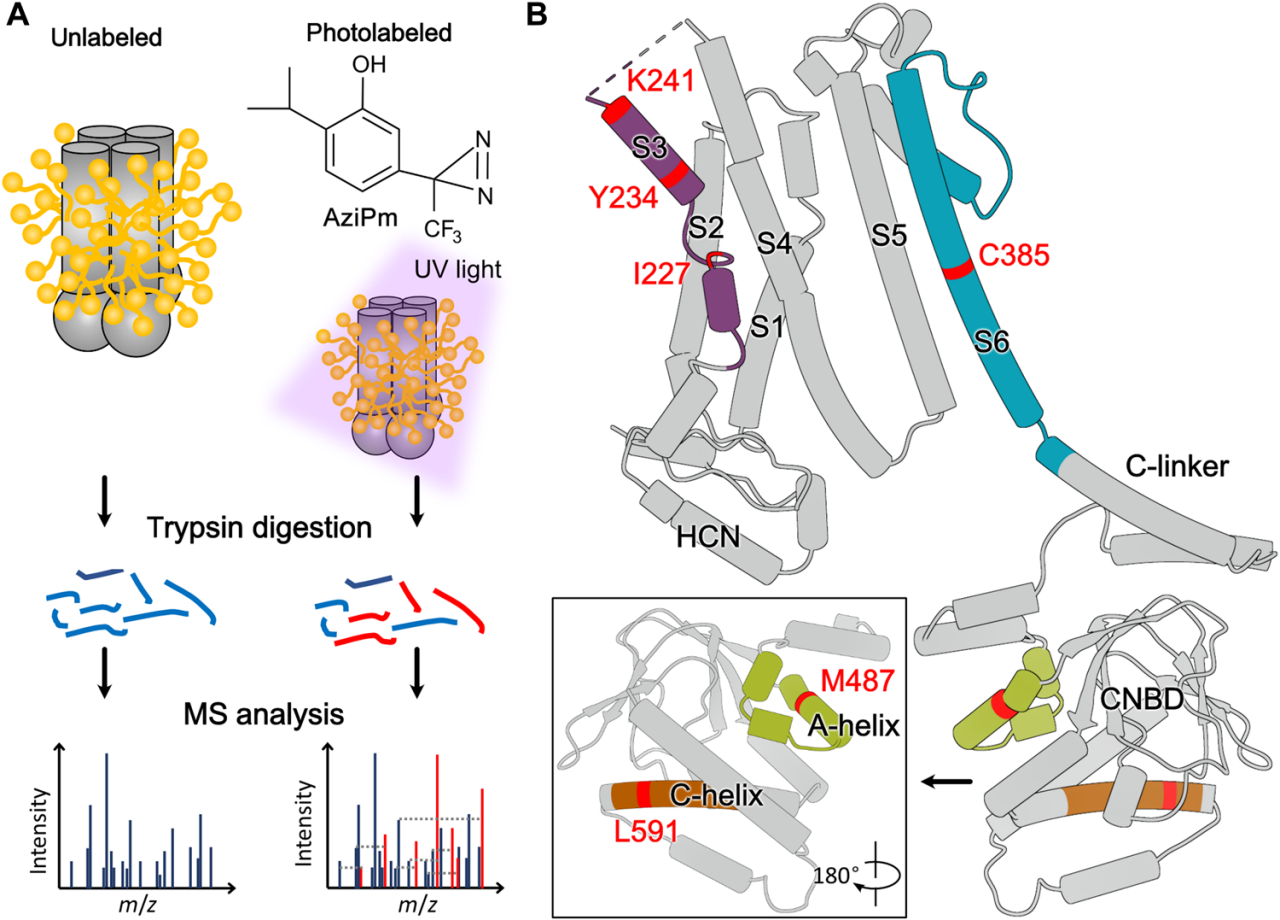
PAL of purified hHCN1 isoform. (**A**) Schematic of the workflow for PAL. Reference mass spectra were obtained using middle-down MS of HCN1 solubilized in digitonin and digested with trypsin. PAL was performed by exposing the digitonin-solubilized HCN1 to UV light in the presence of AziPm (top right). Photolabeled peptides were identified in mass spectra on the basis of their predicted *m*/*z* ratio, and the labeled residues were identified by the additional mass of the AziPm adduct on features in the fragment ion spectra. (**B**) Tubular representation of the structure of the HCN1 subunit highlighting (in red) the positions of the six residues that were photolabeled. Note that either C385 or Y386 is photolabeled, but for clarity, we only show the C385 position. The inset shows another view of the photolabeled residues in the CNBD.

To localize the binding site of propofol in HCN1 channels, we used a well-established PAL approach in conjunction with liquid chromatography with tandem mass spectrometry (LC-MS/MS) (Fig. 1A). Briefly, we equilibrated detergent-solubilized, purified HCN1 protein with a propofol-analog photolabeling reagent and then exposed the sample to ultraviolet (UV) light, generating a short-lived reactive intermediate that covalently modifies amino acids at or near the propofol binding site. The labeled protein was then digested with trypsin to generate peptides containing each of the full-length transmembrane domain (TMD) α helices S1 to S6. LC-MS/MS was then used to separate and identify modified peptides and residues by detecting features in the mass spectra and fragmentation spectra that differ from unlabeled features by the exact mass of the predicted adduct. This method requires the photolabeling analog to have photochemical and functional properties that make it likely to label functionally relevant propofol binding sites. Specifically, the photolabeling reagent should (i) resemble propofol closely enough that it binds in the same location, (ii) generate a photoreactive intermediate with a sufficiently short half-life to preclude diffusion before adduct formation, and (iii) label any amino acid residue in an unbiased manner. In this study, we used *m*-azipropofol (AziPm), a propofol analog containing a trifluoromethyl-diazirine in the meta position on the aromatic ring (Fig. 1) (*22*). Trifluoromethyl-diazirines are efficiently photolyzed at wavelengths (~350 nm) that are not damaging to protein, generating a short-lived carbene intermediate that can react with C–H, O–H, or N–H bonds either in the peptide backbone or any amino acid side chain (*22*, *23*). As required, AziPm replicates the inhibitory effects of propofol on HCN1 currents, shifting the activation curve toward more negative potentials (fig. S1). AziPm is less efficacious and has lower potency than propofol, suggesting that it interacts with overlapping but not identical residues in the propofol binding site. To ensure a comprehensive and unbiased analysis of putative binding sites, it is also requisite that the protein be labeled in a native conformation and under conditions that allow extensive sequence identification. Several detergents were screened, and we found that digestion and analysis of digitonin-solubilized HCN1 yielded the highest sequence coverage: 75%, including TMDs S1 to S4 and S6 and A′ to F′ helices of the cyclic nucleotide–binding domain (CNBD; fig. S2). The N-terminal residues of the S5 peptide were identified in some but not all digests. Our inability to detect the full-length S5 TMD may be due to the presence of a glycosylation sequon (NDS) at the C-terminal end of the predicted S5 tryptic peptide. Given the poor detection of S5, our data do not address the labeling of a possible binding site adjacent to S5. Digitonin-solubilized HCN1 retains a tetrameric structure and has been used to obtain high-resolution cryo–electron microscopy (cryo-EM) structures of this channel, indicating that it retains a native (closed) conformation (*24*).

MS analysis of samples photocrosslinked with AziPm (3 to 100 μM) identified four photolabeled peptides. These peptides met our predetermined criteria for a photolabeled peptide: (i) clearly defined charge states, (ii) mass accuracy <20 parts per million (ppm) from the theoretical mass/charge ratio (*m*/*z*), (iii) delayed chromatographic retention time compared to the unlabeled peptide (effect of the hydrophobic adduct), and (iv) site-defining fragment ions for the adduct with mass accuracy <20 ppm (*25*). The efficiency of photolabeling of each peptide as a function of AziPm concentration for each peptide is shown in Table 1. We used the concentration dependence of labeling efficiency as an indicator of AziPm binding affinity. Notably, the absolute values of labeling efficiency are influenced by multiple factors [site occupancy, photochemistry, and proximity of the reactive carbene to the labeled residue(s)] and are thus not useful for comparing binding between different labeled peptides.

**Table 1.** Photoaffinity-labeled HCN1 peptides identified using MS/MS. AziPm denotes photolabeled residues. [CY]AziPm, either C385 or Y396 is photolabeled. ND, not detected.

Location	Photolabeled peptides	Concentration (μM)	Photolabeling efficiency (%)	
			Singly labeled peptides	Doubly labeled peptides
S3	^220^SWFVVDFISSIPVDYIFLIVE**K**^**AziPm**^GMDSEVYK^249^	100	18.6	1.4
	^220^SWFVVDF**I**^**AziPm**^SSIPVDYIFLIVEK^241^			
	^220^SWFVVDFISSIPVD**Y**^**AziPm**^IFLIVEK^241^			
	^220^SWFVVDF**I**^**AziPm**^SSIPVD**Y**^**AziPm**^IFLIVEK^241^			
	^220^SWFVVDF**I**^**AziPm**^SSIPVDYIFLIVEK^241^	30	18.0	2.3
	^220^SWFVVDFISSIPVD**Y**^**AziPm**^IFLIVEK^241^	10	15.9	1.1
	^220^SWFVVDF**I**^**AziPm**^SSIPVD**Y**^**AziPm**^IFLIVEK^241^			
	^220^SWFVVDFISSIPVD**Y**^**AziPm**^IFLIVEK^241^	3	3.0	ND
S6	^352^AMSHMLCIGYGAQAPVSMSDLWITMLSMIVGAT**[CY]**^**AziPm**^AMFVGHATALIQSLDSSR^404^	10	28.2	ND
		3	ND	
A-helix (CNBD)	^469^LVATMPLFANADPNFVTA**M**^**AziPm**^LSK^490^	100	18.6	ND
		30	6.9	
		10	4.2	
		3	1.0	
C-helix (CNBD)	^582^AFETVAIDR**L**^**AziPm**^DR^593^	100	0.7	ND
		30	0.6	
		10	0.2	
		3	ND	

**Fig. 2.**
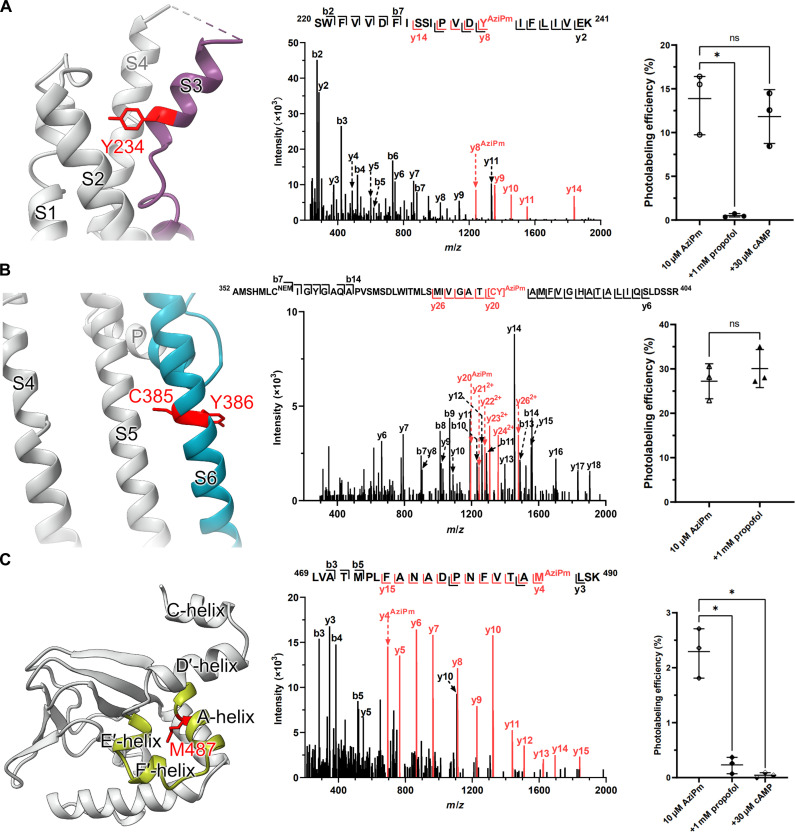
Three distinct AziPm-labeled sites in the HCN1 channel. (**A**) (Left) Structural representation of the VSD highlighting the photolabeled peptide in mauve and the photolabeled Y234 residue in red. (Middle) Fragment ion spectrum of the S3 peptide labeled with 10 μM AziPm; unlabeled fragment ions are shown in black and labeled ions are shown in red. The unlabeled y7 and labeled y8 fragment ions define Y234 as the adducted residue. (Right) Photolabeling efficiencies of the singly labeled S3 peptide in the presence and absence of propofol (1 mM) or cAMP (30 μM). Propofol but not cAMP prevents photolabeling (*n* = 3; one-way ANOVA, *F* = 20.69; Dunnett’s multiple comparison of the means, control versus propofol, *P = 0.002). ns, not significant. (**B**) (Left) Structural representation of the two pore helices (S5 and S6) highlighting the photolabeled peptide in turquoise and photolabeled residues (either C385 or Y386) in red. (Middle) Fragment ion spectrum of the S6 peptide labeled with 10 μM AziPm. The unlabeled y18 (black) and labeled y20 (red) fragment ions localize the adducted residue to either C385 or Y386. (Right) Photolabeling efficiencies of the S6 peptide in the presence and absence of propofol (1 mM). Propofol does not prevent AziPm labeling (*n* = 3; *t* test; *P* = 0.42). (**C**) (Left) Structural representation of the CNBD highlighting the photolabeled peptides in citrine and photolabeled residue M487 in red. (Middle) Fragment ion spectrum of the A-helix peptide of the CNBD labeled with 30 μM AziPm. The unlabeled y3 (black) fragment ion and labeled y4 (red) fragment ion define the adducted residue as M487. (Right) Photolabeling efficiencies of the photolabeled A-helix peptide in the presence and absence of propofol (1 mM) or cAMP (30 μM). Both propofol and cAMP prevent AziPm labeling (*n* = 3 for each sample; one-way ANOVA, *F* = 60.69; Dunnett’s multiple comparison of the means, *P < 0.001).

A peptide containing the S3 helix of the VSD with a single AziPm adduct was identified in samples labeled with 3 μM AziPm. Fragment ion spectra of the singly modified peptide in the sample labeled with 3 μM AziPm identified the adducted residue as Y234 (Fig. 2A). At higher labeling concentrations, additional residues in S3 were labeled, and doubly labeled S3 peptides were detected (fig. S3). At 10 and 30 μM AziPm, the singly and doubly labeled features in the mass spectra contained a mixture of peptides labeled at I227, Y234, or both (fig. S4A). The doubly labeled features from the samples labeled with 30 and 100 μM AziPm revealed labeling at I227, Y234, and an additional residue near the C terminus of S3. Fragment ion spectra of peptides singly labeled with 100 μM AziPm supported an adduct at the C terminus localized to K241 (fig. S4B). Collectively, these data indicate that Y234 is labeled with the highest affinity but that additional nearby residues are labeled at higher AziPm concentrations. While the additional labeled residues on S3 may represent independent low-affinity binding sites or residues labeled by ligand diffusing away from its binding site, we focused on the Y234 residue because it was labeled at pharmacologically relevant concentrations. To confirm that AziPm labeling of Y234 identifies a specific propofol binding site, we examined the ability of an excess of propofol to prevent AziPm labeling. Labeling with 10 μM AziPm was almost entirely prevented by a 100-fold excess of propofol [Fig. 2A; one-way analysis of variance (ANOVA), *F* = 20.69; Dunnett’s multiple comparison of the means, control versus propofol, *P* = 0.002], consistent with a specific propofol binding site near Y234. A saturating (30 μM) cAMP concentration had no effect on AziPm labeling of the S3 peptide (Fig. 2A; Dunnett’s multiple comparison of the means, control versus cAMP, *P* = 0.65).

A peptide containing the S6 transmembrane helix with a single AziPm adduct was detected following labeling with 10 μM but not 3 μM AziPm. We did not consistently detect unlabeled and labeled S6 peptides at 30 or 100 μM AziPm and thus did not report labeling efficiency at these concentrations (Table 1). The S6 peptide is challenging to detect and quantitate because it is long (52 residues) and poorly charged, making it difficult to ionize, and because it contains two cysteine residues within the hydrophobic α helices; these cysteines are difficult to fully reduce and alkylate, and the S6 peptide signal can thus be broken up into multiple features in a mass spectrum. Fragment ion spectra indicate that the S6 peptide is labeled at either C385 or Y386 (Fig. 2B). A 100-fold excess of propofol did not substantially inhibit the labeling of the S6 peptide (Fig. 2B; *t* test, *P* = 0.42). While the absence of propofol inhibition might indicate that this is a site to which AziPm binds but propofol does not, the AziPm labeling of S6 is consistent with recently published cryo-EM data indicating a propofol binding site in the pore region (*26*). The absence of competitive prevention of labeling with propofol may be either because AziPm binds to this site with higher affinity than propofol or because AziPm and propofol bind in a common pocket but with insufficient overlap to allow competitive prevention of labeling. During the review process, we were asked to validate the absence of labeling of S6 with 3 μM AziPm. Using a mass spectrometer with higher sensitivity, we were able to detect labeling of S6 (~1.5% efficiency of labeling) but confirmed that labeling was not prevented by 1 mM propofol. These data indicate that AziPm labels a probable propofol binding site in S6 and that propofol binds to this site with comparable or lower affinity than to the voltage-sensor site.

Two photolabeled peptides were detected in the CNBD: an A-helix peptide labeled at M487 (Fig. 2C) and a distal C-helix peptide labeled at L591 (fig. S5). The labeling efficiency of both peptides was linear (nonsaturating) over the tested concentration range of AziPm (3 to 100 μM) (Table 1), suggesting that the labeled residues contribute to low-affinity binding sites. Consistent with this, labeling of the A-helix peptide (M487) was substantially inhibited by a 100-fold excess of propofol (Fig. 2C, *n* = 3; one-way ANOVA, *F* = 60.69; Dunnett’s multiple comparison of the means, *P < 0.001), clarifying that it does contribute to a low-affinity propofol binding site. Labeling was also prevented by 30 μM cAMP (Fig. 2C, *n* = 3; one-way ANOVA, *F* = 60.69; Dunnett’s multiple comparison of the means, *P < 0.001), consistent with either competitive or negative allosteric modulation of propofol binding. The C-helix peptide (L591) was labeled with very low efficiency, and the low signal intensity of the labeled peptide precluded evaluation of the effects of excess propofol.

### Atomistic MD simulations of propofol binding to the HCN VSD

**Fig. 3.**
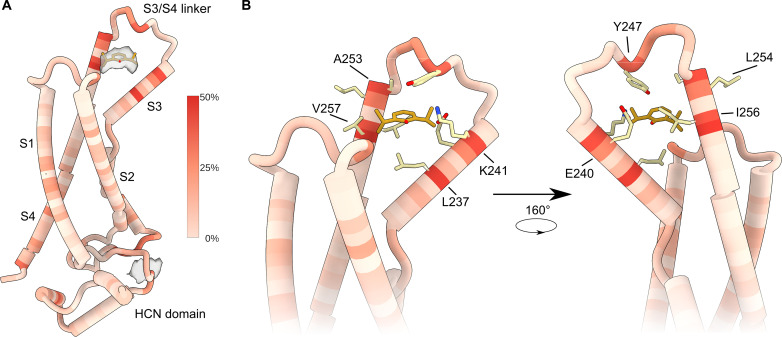
MD simulation identifies a binding pocket delimited by transmembrane helices S3 and S4. (**A**) Three-dimensional heatmap representing averaged propofol occupancy on VSD and HCN domain structure of HCN1 in the closed conformation. The occupancies from MD trajectories were calculated using PyLipID. Gray density depicts the time-averaged propofol density at a threshold of 0.06 plotted relative to the VSD structure. (**B**) Close-up views of the heatmap shown in (A) highlighting the region with the highest occupancies. The side chains of residues predicted to interact with propofol (orange) in the binding pocket are shown as sticks.

To validate and probe the plausibility of the propofol binding sites identified by PAL, we conducted a propofol flooding experiment using all-atom MD simulations on the closed-state HCN1 structure [Protein Data Bank (PDB): 5U6O]. In a flooding experiment, the ligand is added at high concentrations to improve sampling and increase the likelihood of identifying the correct binding pocket and pose (*27*). The MD simulations focused on the site(s) in the VSD containing transmembrane helices S1 to S4. In the simulations, we pre-equilibrated the system with 100 mM propofol and simulated two sets of 500 ns. Mapping the time-averaged propofol density onto the VSD shows clear localization at the upper crevice near the S3-S4 paddle motif, close to the photolabeling sites on S3 (Fig. 3A). The localized density at the top of the S3-S4 paddle motif was absent in flooding experiments on the activated VSD structure (*28*) (PDB: 6UQF), likely because the voltage sensor is in the down position, and thus, the putative propofol binding pocket is not formed (fig. S6). These findings are consistent with inhibition mediated by preferential binding to and stabilization of the closed state (*11*).

To further refine the propofol binding site, we used PyLipID, a software program that determines representative binding poses by evaluating all bound poses on the basis of a scoring function (*29*). PyLipID uses a dual cutoff distance method to identify interacting residues. This approach aims to mitigate sudden disruption in interaction because of the “rattling in a cage” effect. The dual cutoff distance defines the boundaries under which propofol is considered to interact with the protein continuously. We set the boundaries such that propofol is considered bound to a protein residue if any of its atoms are within a distance of 2.5 Å to any atom of the interacting residue in the protein. Once the distance exceeds 3.5 Å, propofol is considered unbound. The per-residue occupancy mapped onto the VSD structure shows a hotspot near the S3-S4 paddle site implicated by the density analysis above (Fig. 3A). The representative binding pose of propofol at this site fits well into the time-averaged density, illustrating stable binding during the duplicate trajectories. The occupancy heatmap in Fig. 3B illustrates that propofol predominantly interacts with residues L237 and K241 in the S3 transmembrane helix while also engaging with residues A253, L254, I256, and V257 in the S4 transmembrane helix throughout 30 to 50% of the trajectory. Moreover, the simulations showed that residue Y247 at the S3/S4 linker coordinates a π-π interaction with propofol. Among these residues, L237 (9%) and V257 (47.2%) showed the highest contact occupancy. Our simulations indicate that the interactions are predominantly hydrophobic in nature, likely established by the isopropyl groups of propofol. Although we observed the partitioning of propofol into the membrane, the simulations suggest that propofol enters the binding pocket primarily from the aqueous phase through portals exposed to the extracellular side (fig. S7).

Although propofol binds to the S3/S4 linker site in many poses, the top three poses ranked by PyLipID analysis are nearly identical (fig. S8A). Starting from these three poses, which also represent different protein conformations, we performed docking to a box (20 Å by 20 Å by 30 Å) centered around the identified site for both propofol and the AziPm derivative. Propofol docking poses show good general agreement with the atomistic flooding results, with the top 15 poses (5 poses from each of the 3 starting points) from docking correlating highly with the time-averaged propofol density in the simulations (fig. S8B). The top scoring poses for propofol docking are close to the site identified by PyLipID; however, the ring is flipped nearly 180° for all the top-ranked docking poses, with the hydroxyl group pointing back toward the S4 helix (fig. S8C). The ensemble of docking poses for AziPm resembles that of propofol (fig. S8B), but the top poses for AziPm are more diverse (fig. S8D). One of these top poses for AziPm extends further down into the VSD crevice. This brings the photolabile group of AziPm into closer proximity to Y234 while still maintaining overlap with the propofol site.

### Functional analysis of a putative propofol binding pocket

**Fig. 4.**
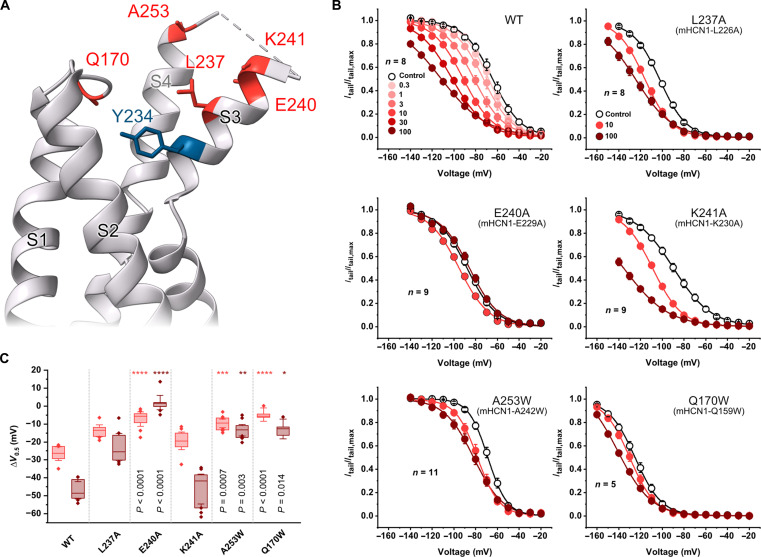
Functional evaluation of key residues in the putative propofol binding pocket. (**A**) Close-up ribbon representation of the structure of HCN1 VSD. The residues marked in red were tested for their role in mediating propofol modulation of HCN channel gating. The photoaffinity-tagged residue Y234 is shown in blue. (**B**) Conductance-voltage curves of the WT and mutant mHCN1 channels obtained in the presence of various concentrations of propofol. Control conductance-voltage curves were obtained without propofol (unfilled symbols). The mutants are numbered according to their equivalent position in hHCN1, and the corresponding residue positions in mHCN1 are in parentheses below. The data shown represent means ± SEM. (**C**) Box plot of propofol-induced shifts of *V*_0.5_ of channel activation for WT and mutant channels. The shifts in *V*_0.5_ are plotted relative to the control recordings for two concentrations of propofol: 10 μM (light red) and 100 μM (deep red). For each concentration, the change in mean half-maximal activation was compared to WT using a nonparametric Kruskal-Wallis post hoc test with a significance level of *P* < 0.05. ****P < 0.0001; ***P < 0.001; **P < 0.01; *P < 0.05.

To validate the putative propofol binding site in the S3-S4 region and evaluate the functional contribution of individual side chains, we tested the effects of mutations in this binding pocket on propofol modulation. The photolabeling experiments identified residue Y234 in the VSD as a contributor to a potential propofol binding site, but the Y234A-HCN1 mutant did not express in *Xenopus* oocytes and, therefore, could not be tested. Since MD simulations show that Y234 marks the lower boundary of a water-filled cavity, we explored the contribution of other residues in the S3 TMD within this putative binding pocket. In the wild-type (WT) mouse HCN1 (mHCN1), the normalized conductance-voltage (*G*-*V*) curve is left shifted by 20 mV at 10 μM propofol and shifts an additional 20 mV at 100 μM concentration (Fig. 4, B and C, and table S1). Substituting either L237 or E240 with alanine substantially reduced the inhibitory effect of propofol. In 10 μM propofol, the *G*-*V* curve shifts by 14 mV in the L237A mutant, whereas it shifts by only 7 mV in the E240A mutant (Fig. 4C). The K241A mutant channel, despite being within the high-occupancy zone in MD simulations, remains as sensitive as WT HCN1 (Fig. 4, B and C).

Next, we tested two additional sites within or near the binding pocket: A253 on top of the S4 segment and Q170 on the S1-S2 loop. A253 was unexpectedly identified as a high-occupancy site in the analysis of MD trajectories, but we wondered whether its small size is necessary for defining the binding pocket. Substitution of alanine with tryptophan (A253W) resulted in a substantial reduction in propofol-mediated inhibition. Propofol (10 μM) causes an 8-mV shift in half-maximal activation of the *G*-*V* curve. MD trajectories also show that Q170, which is in the flexible S1/S2 linker, interacts weakly with propofol. To test whether substitutions in this location have any effect on propofol modulation, we substituted tryptophan at this position. The Q170W mutation markedly shifts the *G*-*V* curve to −125 mV, and adding 10 μM propofol further shifts this curve by only another 4 mV. One of the characteristic features of propofol modulation of WT channels is the slowed activation kinetics, which is also observed in the Q170W mutant, suggesting that the tryptophan at this position may act as an intrinsic propofol analog and prevent binding of exogenously added propofol (Fig. 4 and table S1).

## DISCUSSION

Propofol is a widely used alkylphenol general anesthetic. In addition to its hypnotic action, which is mediated by potentiating the activity of inhibitory GABA_A_ ionotropic receptors (*30*), propofol has other beneficial effects, presumably because of its proclivity to interact with other membrane receptors (*31*, *32*). Propofol inhibition of the HCN channel mediates antihyperalgesia in animal models of chronic pain (*16*–*18*, *33*), and human studies have shown that subhypnotic doses of propofol are analgesic (*34*, *35*). While HCN channels are an attractive target for developing analgesic agents, a clinically useful propofol analog targeting HCN channels should (i) preferentially inhibit HCN1 rather than the HCN2 or HCN4 isoforms, thus reducing potential effects on cardiac automaticity, and (ii) minimize potentiation of GABA_A_-mediated currents, thus limiting its sedative effect. The rational design of such a propofol analog will be greatly facilitated by identifying the propofol binding site(s) on HCN1 channels.

Identification of the propofol binding sites in the HCN channel has remained challenging for several reasons. First, structure-function measurements and mutational analyses, such as those used to identify local anesthetic binding sites in voltage-gated sodium channels, can only provide an indirect measure of binding. In such studies, it is impossible to completely distinguish between the effects of a mutation on binding as opposed to gating (“binding-gating problem”) (*36*). Second, the binding affinity of propofol is too low for direct binding assays that depend on the separation of free ligands from the bound ligands, and its hydrophobicity makes it difficult to discriminate between the bound versus lipid-partitioned fraction in scintillation proximity assays, which can be used for hydrophilic ligands with modest binding affinities (*37*). Last, the chemical structure of propofol is close to an aromatic amino acid, which makes it challenging to resolve the bound drug in cryo-EM structures with a high degree of confidence at the current resolution of HCN1 structures (*24*, *28*, *38*, *39*).

In this study, we use a combination of PAL and MD simulation to identify the propofol binding pocket in human HCN1 (hHCN1). Our approach is inspired by previous PAL studies mapping the propofol binding site(s) on the GABA_A_ receptor using either *o*-propofol diazirine (*o*-PD) or AziPm (*25*, *40*, *41*). *o*-PD best mimics the action of propofol on GABA_A_ receptors, whereas AziPm has superior photochemical properties (shorter lifetime of photoactive intermediate and absence of amino acid–specific labeling). In this study, we used AziPm because it both mimics propofol’s inhibition of HCN1 activity and is effective at photolabeling the HCN1 channel.

Using this approach, we identified a peptide containing the S3 helix of the VSD in which the Y234 residue was labeled with 3 μM AziPm. Additional S3 residues near Y234 were labeled at higher (10 to 100 μM) AziPm concentrations. Y234 labeling with 3 or 10 μM AziPm was prevented by a 100-fold excess of propofol, consistent with a specific binding site near this residue. Identification of a propofol binding site near Y234 was supported by propofol flooding experiments in atomistic MD simulations of the VSD in a lipid bilayer, which showed that propofol is localized one helical turn above Y234 in a binding pocket defined by the extracellular ends of the S3 and S4 helices. Notably, the time-averaged propofol densities are observed in the VSD in the resting conformation but not the activated conformation. Although propofol molecules reside in the binding pocket for a large fraction of the simulation time, they can bind in more than one pose, consistent with their small size and modest affinity. Last, site-directed mutagenesis of the residues in the binding pocket identified using MD and PAL, coupled with functional measurements, shows that single amino acid substitutions can abrogate the shifts in voltage dependence even at concentrations as high as 100 μM. Collectively, these data indicate that propofol inhibition of HCN1 activation is mediated by a specific binding site in the VSD.

While unexpected, it is not without precedent that AziPm labels a residue (Y234) that is one to two helical turns away from the binding pocket predicted by flooding simulations. A similar difference between propofol and AziPm binding sites is also observed in GABA_A_-R: I239, the residue most prominently labeled by AziPm in the canonical b3/a1 intersubunit propofol binding site (*25*, *40*), is one to two turns below where propofol is shown to bind in a cryo-EM structure of the protein (*42*). AziPm labeling is prevented by excess propofol, consistent with overlapping sites. As in HCN, AziPm is less potent and efficacious than propofol at modulating the channel function.

Our AziPm and propofol docking studies in HCN1 indicate that the lowest-energy poses for AziPm and propofol are slightly different in the VSD pocket, but there is sufficient overlap that the binding of one would preclude the binding of the other. The photoreactive diazirine group of AziPm is positioned close to Y234, even though this residue is not within the propofol binding pocket in the docking or MD simulations. We note that although the docking and MD simulations for propofol show slight discrepancies regarding the binding pose, the pose from flooding simulations is considered more reliable. The explicit modeling of water and lipids makes atomistic MD simulations the preferred method for small, hydrophobic molecules like propofol and other anesthetics (*43*).

Nevertheless, it is important to remember that the binding pocket prediction in our simulations reflects the most stable interactions on the basis of static or semistatic conformations. However, transient interactions or conformational shifts that are not fully captured in simulations may occur in experiments like photolabeling, allowing ligands to access nearby residues outside the predicted binding site. These differences further highlight the importance of combining simulation data with experimental validation to capture the full range of protein-ligand interactions.

Two labeled peptides in the CNBD were also identified by AziPm photolabeling. While CNBD labeling was prevented by both propofol and cAMP, we did not further evaluate this site because HCN1 constructs in which the CNBD has been deleted retain sensitivity to propofol (*21*). Thus, it is unlikely that propofol’s inhibitory effect is mediated via the CNBD region.

We also identified a labeled peptide in the S6 helix of the pore domain in which AziPm adduction was narrowed to either the C385 or Y386 residue. Labeling of the S6 peptide was not prevented by a 100-fold excess of propofol. While this manuscript was under consideration, a cryo-EM structure of nanodisc-reconstituted HCN1 in the presence of 1 mM propofol was published (*26*). This structure showed additional density presumably corresponding to propofol near the AziPm-adducted C385/Y386 residues but none corresponding to a propofol site in the VSD. There are a couple of possible reasons for the lack of density at that site: (i) the S3-S4 loop that is part of the VSD binding pocket for propofol is not resolved in the structures, and (ii) the addition of 3 mM fos-choline before plunge freezing may have extracted the propofol from the water-accessible VSD site but not the lipid-exposed pore site. Admittedly, these are speculations, and at this point of time, we do not know the reasons for the lack of propofol densities in the HCN1 VSD.

Our results, combined with the recently reported cryo-EM data, indicate that there are likely two distinct propofol binding sites in HCN1: one in the VSD and another in the pore domain. Mutagenesis of M305 or T384, which constitutes the propofol binding site identified in the pore, reduces but does not abrogate the hyperpolarizing shift in the voltage dependence of channel opening elicited by propofol. In contrast, selected mutations in the VSD site eliminate this effect of propofol. In addition to producing a hyperpolarizing shift in channel activation, high concentrations of propofol also decrease the maximum HCN current (*19*, *21*). Tibbs and colleagues have shown that propofol does not decrease single-channel conductance but reduces the maximum open probability at saturating propofol concentrations (*21*). It is thus plausible that propofol binding in the pore region near the S6-labeled residue (C385 or Y386) either blocks the channel pore or stabilizes its closed conformation.

A notable feature of previously identified propofol binding sites is that they are all contained within the TMDs and are either accessible via the lipid membrane or are lipid facing. For example, in the structure of the pentameric ligand-gated ion channel GLIC, propofol is bound to an intrasubunit pocket at the extracellular end of the transmembrane helices, a site that overlaps with the pocket identified using PAL (*44*). In heteromeric GABA_A_ channels, multiple propofol binding sites have been mapped to the interfaces of the b-a subunits using AziPm and an intrasubunit pocket using *o*-PD. Cryo-EM structures of a1b2g2 GABA_A_ receptors in the presence of propofol reveal densities at b-a interfaces corresponding to one of the sites identified using PAL (*41*, *42*). In stark contrast, the propofol site in the voltage-sensor region of the HCN1 channel faces the extracellular solution, and the bound drug is unlikely to interact with or gain access to the lipid membrane directly. On the basis of these observations, we surmise that propofol enters the binding pocket from the aqueous phase rather than partitioning into the membrane and laterally diffusing to its binding site. This property may be exploited to develop a water-soluble propofol analog that selectively inhibits peripheral HCN1 channels. Together, these findings lay the groundwork for developing more potent and specific HCN1 modulators that mimic propofol action and, therefore, serve as targeted analgesics for pain treatments.

## MATERIALS AND METHODS

### Constructs and molecular biology

We used the hHCN1 single-molecule (hHCN1-SM) construct for PAL studies as described previously (*45*). The mHCN1 construct was used for electrophysiological measurements because these channels elicit robust tail currents that can be easily resolved from the capacity transients in the two-electrode voltage clamp (TEVC) recordings. Site-directed mutagenesis was performed using the FastCloning technique (*46*). The primers for mutagenesis are listed in table S2. Cloning resulted in different recombinants because of a GC-rich stretch in the N terminus. We thus modified the backbone by reducing the GC content with silent mutations between nucleotides 75 and 141. The changes in the backbone resolved the recombination issue and facilitated the mutagenesis. All constructs were verified by Sanger sequencing of the entire coding length before use.

### Expression in mammalian cells and protein purification

hHCN1-SM was expressed in suspension cultures of Freestyle HEK cells using a BacMam system. Briefly, the TS-eGFP-hHCN1-SM-pEG plasmid was transformed to DH10Bac competent cells (Bac-to-Bac; Invitrogen) to produce recombinant bacmid DNA. The bacmid DNA containing this construct was selected by blue/white selection, amplified, and transfected into Sf9 cells (1 to 2 μg/100 cells) using Cellfectin II (Invitrogen). The supernatant containing P1 baculoviruses was harvested after 5 to 7 days, sterile filtered, and used to generate P2 baculovirus at a dilution factor of 100. The baculovirus was then supplemented with 2.5% fetal bovine serum before use. For transduction, an 800-ml suspension culture of Freestyle HEK cells (3 × 10^6^ to 3.5 × 10^6^ cells/ml) was transduced with 3.5 to 5% baculovirus. After 10 to 12 hours at 37°C, the cells were supplemented with 10 mM sodium butyrate and kept at 30°C for another 48 to 50 hours. The cells were harvested with low-speed centrifugation, and cell pellets were stored at −80°C. All steps involved in protein purification were conducted at 4°C. Cells were lysed by sonication in a hypotonic lysis buffer [20 mM KCl, 10 mM tris(hydroxymethyl)aminomethane (tris), and protease inhibitor cocktail (P8340, Sigma-Aldrich), pH 8.0]. Membranes were collected by centrifugation at 50,000*g* for 45 min and solubilized in solubilization buffer [300 mM KCl, 40 mM tris, 4 mM dithiothreitol (DTT), 10 mM lauryl maltose neopentyl glycol, 2 mM cholesterol hemisuccinate, and protease inhibitor cocktail (P8340, Sigma-Aldrich), pH 8.0] for 1.5 hours under continuous rotation at 12 rpm. The solubilized protein was collected at 50,000*g* for 45 min and incubated for 2.5 hours with Strep-Tactin Sepharose resin (2-1201-025, IBA) that was pre-equilibrated in wash buffer (300 mM KCl, 20 mM tris, 2 mM DTT, and 0.05% digitonin, pH 8.0). The resin-sample mixture was then loaded onto a column and eluted with wash buffer containing 10 mM *d*-desthiobiotin (D1411, Sigma-Aldrich). The sample was incubated with 3C protease overnight at 4°C to remove the GFP (green fluorescent protein) tag. The sample was concentrated to a maximum final concentration of 8 mg/ml by using a 100-kDa Amicon centrifugal filter tube before being subjected to size exclusion chromatography (SEC). For this, the sample was loaded onto a Superose 6 10/300 GL column (29-0915-96, Cytiva) that was pre-equilibrated with SEC buffer (50 mM KCl, 20 mM tris, 2 mM DTT, and 0.05% digitonin, pH 8.0). The fractions corresponding to the peak of the protein’s tetrameric assembly were pooled and concentrated to a range of 1 to 1.5 mg/ml. Subsequently, the sample was subject to photolabeling or preparation for MS analysis.

### Heterologous expression and electrophysiology

WT mHCN1 and mutant currents were expressed in *Xenopus laevis* oocytes and recorded using TEVC as previously described (*47*). Briefly, isolated oocytes were injected with 40 to 50 ng of complementary RNA and incubated at 17°C for 24 to 48 hours before recording. All TEVC recordings were obtained at room temperature. The currents were recorded using a CA-1B amplifier (Dagan) at a sampling frequency of 10 kHz and were filtered with a cutoff frequency of 5 kHz. Electrodes were fabricated from thin-walled glass pipettes (World Precision Instruments) using a P97 micropipette puller (Sutter Instruments). The pipette resistance was in the range of 0.5 to 1 megohm using 3 M KCl. The bath solution contained 107 mM NaCl, 5 mM KCl, 20 mM Hepes, and 2 mM MgCl_2_ (pH 7.4 NaOH). Conductance-voltage curves were obtained by measuring the tail currents at 0 mV elicited by pulsing to voltages ranging from −20 to −140 mV for 2 s from a holding potential of −10 mV. The peak amplitude of the tail current reflects the fraction of activated channels and was plotted as a function of the test pulse. The activation curve was normalized to the maximum tail current and fitted with a single Boltzmann function

$$\frac{G}{G_{\text{max}}}=\frac{1}{1+e^{\frac{\left(V-V_{0.5}\right)}{k}}}$$

where V is the conditioning pulse, $V_{0.5}$ is the voltage at which 50% of the channels are open, and *k* is the inverse slope factor. Data were collected and analyzed using pCLAMP10.0 (Molecular Devices) and plotted using OriginPro 2020b (OriginLab Corp., Northampton, MA).

### Photolabeling and middle-down MS

For photolabeling experiments, 100 to 150 μg of purified hHCN1-SM protein (in 50 mM KCl, 20 mM tris, 2 mM DTT, and 0.05% digitonin, pH 8.0) was incubated for 1 hour at 4°C either alone (control) or with varying concentrations (3 to 100 μM) of AziPm, a photoactive analog of propofol. AziPm was synthesized according to the literature (*22*) and was added to the protein from concentrated ethanolic stock such that the final ethanol concentration was <1% and was equal in all samples. For experiments examining competitive prevention of photolabeling, membranes were incubated at 4°C for 1 hour with AziPm in the presence or absence of a putative competitor (1 mM propofol or 30 μM cAMP); an equal volume of vehicle (ethanol) was added to all samples (*25*). The samples were then irradiated in a 100-μl quartz cuvette for 5 min using a photoreactor emitting light at >320 nm as previously described (*48*). Following UV irradiation, the samples were exchanged to a 50 mM triethylammonium bicarbonate, 0.05% digitonin, pH 7.5 buffer using Biospin6 columns (Bio-Rad). The HCN1 protein was then reduced with 5 mM tris(2-carboxyethyl)phosphine for 30 min, alkylated with 5 mM *N*-ethylmaleimide (NEM) for 45 min in the dark, and quenched with 8 mM DTT for 15 min. These three steps were done at room temperature. Samples were then digested by adding 10 μg of trypsin per 100 mg of protein and incubating the samples for 5 days at 4°C. The digestions were terminated by adding formic acid in a final concentration of 1%, followed directly by LC-MS analysis on an Orbitrap Elite mass spectrometer (Thermo Fisher Scientific). Twenty microliters of samples was injected onto a home-packed PLRP-S (Agilent, Santa Clara, CA) column (12 cm by 75 μm, 300 Å), separated with a 145-min gradient from 1 to 90% acetonitrile, and introduced to the mass spectrometer at 800 nl/min with a nanospray source, as previously described (*25*). The survey MS1 scans were acquired at high resolution (60,000 resolution) in the range of *m/z* = 100 to 2000, and the fragmentation spectra were acquired at 15,000 resolution. Data-dependent acquisition of the top 20 MS1 precursors with exclusion of singly charged precursors was set for MS2 scans. Fragmentation was performed using collision-induced dissociation or higher-energy collisional dissociation (HCD) with a normalized energy of 35%. The data were acquired and reviewed with Xcalibur 2.2 (Thermo Fisher Scientific). A follow-up LC-MS analysis aimed at detecting labeling with 3 μM AziPm was performed on a Dionex U3000 nano-LC coupled to an Orbitrap Ascend Tribrid mass spectrometer. Ten microliters of samples was loaded at a flow rate of 5 μl/min, concentrated, and washed for 3 min on a homemade PLRP-S precolumn (5-μm particle size, 300-Å pore size from Agilent, 75-μm inner diameter, and 2-cm length). Peptides were separated on a homemade PLRP-S column (same packing material as the precolumn, 75-μm inner diameter, and 10-cm length) with a 90-min run-time gradient ranging from 10 to 90% acetonitrile. Data were acquired in data-dependent mode as described above. MS1 spectra were acquired with a cycle time of 3 s and a resolution of 120,000, followed by MS/MS HCD fragmentation with a resolution of 30,000.

The LC-MS data were searched against a customized database containing the sequence of the hHCN1-SM protein using PEAKS Studio 10.6 (Bioinformatics Solutions Inc., Waterloo, ON, Canada). Search parameters (*25*) were set for a precursor mass accuracy of 20 ppm, a fragmentation ion accuracy of 0.1 Da, and a maximum of five missed cleavages on either side of peptides with trypsin digestion. Methionine oxidation, cysteine alkylation with NEM or DTT, and adducts of AziPm (mass, 216.08) on any amino acid were included as variable modifications. Photolabeling efficiency was estimated by measuring the area under the curve (AUC) of selected ion chromatograms of the photolabeled and corresponding nonphotolabeled peptides and calculating the AUC of the photolabeled peptide divided by the sum of the AUC of the photolabeled and nonphotolabeled peptides (×100). The MS2 spectra of photolabeled TMD peptides were also manually analyzed for fragment ion charge state and mass accuracy and to confirm the sequence assignment and sites of adduction.

### MD simulation

The VSD of HCN1 in the resting state (PDB: 5U60) and activated state (PDB: 6UQF), corresponding to residues 94 to 289, was prepared using the CHARMM-GUI (https://charmm-gui.org/) server (*49*, *50*), embedded in a 1-palmytoyl-2-oleoyl-phosphatidyl-choline bilayer (66 Å by 66 Å), and solvated in a 150 mM KCl solution. The CHARMM36 force field was used for the protein, lipids, and ions and the TIP3P model for water (*51*, *52*). The CGenFF module in CHARMM-GUI was used to parameterize propofol, which was inserted into the system at a concentration of roughly 100 mM (17 molecules) using gmx insert-molecules by replacing water molecules in the system before minimization and equilibration (*53*). Energy minimizations and equilibrations were performed according to the default output of CHARMM-GUI aside from the final equilibration with only backbone and propofol restrained for 3 ns. Propofol was restrained throughout the equilibration before the production runs to prevent interactions with the protein during the equilibration steps. The 500-ns duplicate simulations were then performed in the NPT ensemble, maintaining gentle restraints (50 kJ mol^−1^ nm^−2^) on the protein backbone to prevent the unfolding of the HCN domain, which had been reported previously (*54*). Temperature and pressure were maintained at 310 K and 1 bar using the Nose-Hoover thermostat (*55*) and Parrinello-Rahman barostat (*56*), respectively. The particle mesh Ewald algorithm (*57*) was used to calculate long-range electrostatic interactions. A switching function was applied between 10 and 12 Å for the van der Waals interactions. LINCS (*58*) was applied to restrain the bond lengths of hydrogen atoms, allowing an integration time step of 2 fs. Time-averaged propofol densities were calculated using MDAnalysis using a voxel size of 1 Å and averaged across replicates. PyLipID was used to characterize interactions between propofol and protein using a dual cutoff at 2.5-Å lower bound and 3.5-Å upper bound (*29*).

### Docking of propofol and AziPm

Coordinates from propofol were removed from the three binding poses reported by PyLipID, and the remaining protein was subjected to docking with propofol or AziPm using AutoDock Vina using the Webina server (https://durrantlab.pitt.edu/webina/). Docking was performed using a box (20 Å by 20 Å by 30 Å) encompassing the extracellular half of the VSD centered around the propofol binding site from MD simulations. Nine poses were generated for each of the three PyLipID protein conformers, but only five poses per conformer are included in figures for clarity (fig. S8). The remaining poses can be found in the public deposition of the docking coordinates on Zenodo (DOI: 10.5281/zenodo.13771584).
