# Supplementary material for: A propofol binding site in the voltage sensor domain mediates inhibition of HCN1 channel activity
Source: Sci Adv. 2025 Jan 3;11(1):eadr7427. doi: 10.1126/sciadv.adr7427 (PMC11698089; doi:10.1126/sciadv.adr7427)
Supplement: Supplementary file 1 — Figs. S1 to S8 Tables S1 and S2 [file sciadv.adr7427_sm.pdf]

Science Advances
AAAS

Supplementary Materials for

A propofol binding site in the voltage sensor domain mediates inhibition of HCN1 channel activity

Verena Burtscher *et al.*

Corresponding author: Alex S. Evers, eversa@wustl.edu; Baron Chanda, bchanda@wustl.edu

Sci. Adv. **11**, eadr7427 (2025)
DOI: 10.1126/sciadv.adr7427

This PDF file includes:

Figs. S1 to S8
Tables S1 and S2

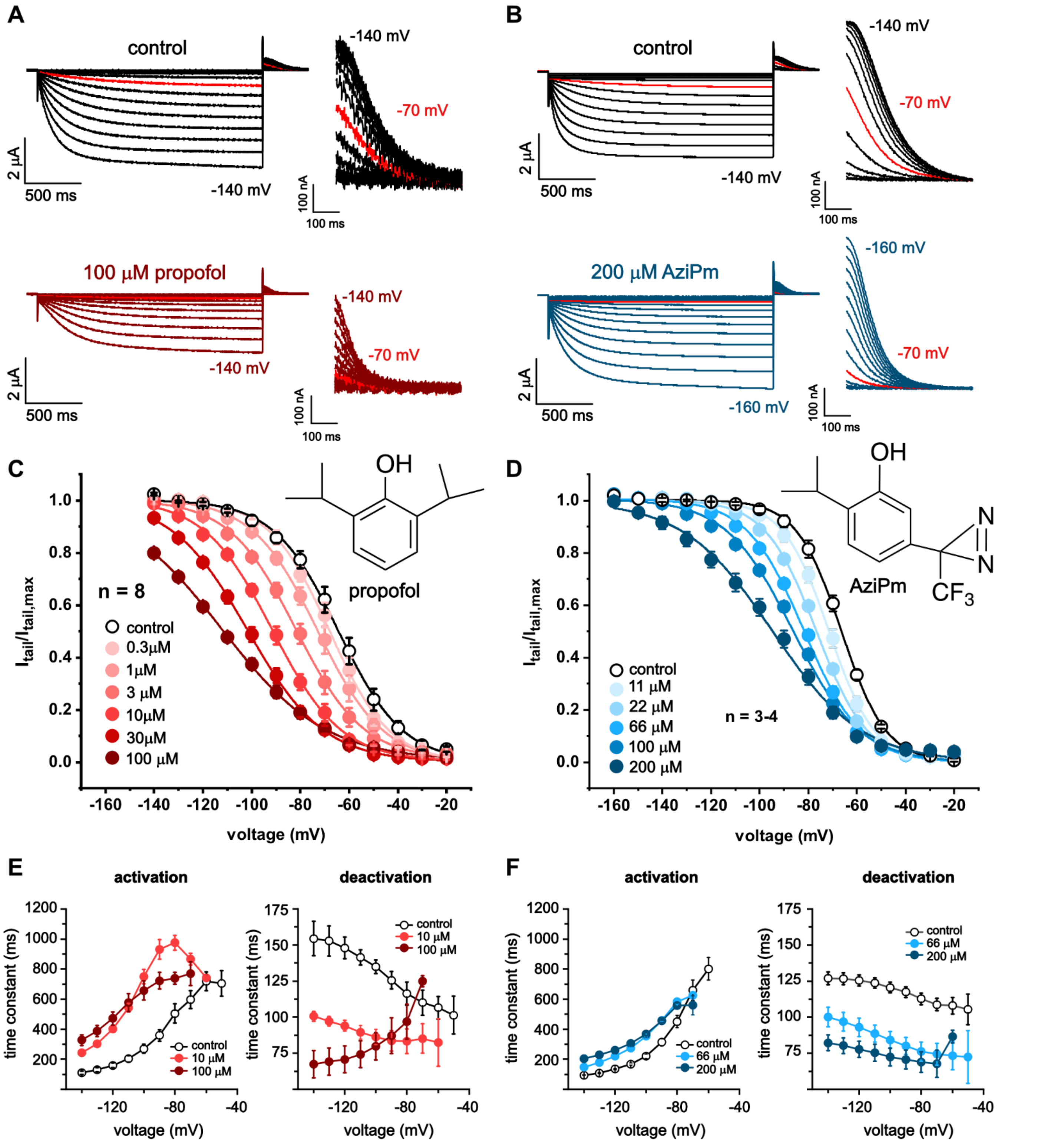

Fig. S1: AziPm shifts the voltage-dependent activation of HCN1 channels to more negative potentials. A-B, Representative family of current traces in the absence (top) and the presence (bottom) of either 100 μM propofol or 200 μM AziPm. Insets: Tail currents of the corresponding recording. Current traces recorded at -70 mV are highlighted in bright red. **C-D,** Conductance-voltage curves obtained in the absence (black circles) and presence of various concentrations of either propofol (red-shaded circles) or AziPm (blue-shaded circles). AziPm shifts the voltage of half-maximal activation in a concentration-dependent manner. The maximal shift of $V_{0.5}$ is -28.4 ± 2.6 mV, while the propofol-mediated shift is -42.7 ± 3.1 mV. Data points are shown as mean ± SEM. Insets: Chemical structure of either propofol or AziPm. **E,** Activation (left panel) and deactivation time constant (right panel) in the absence (black circles) or the presence of either 10 or 100 μM

propofol (red-shaded circles). Propofol increases the activation time constant and decreases the deactivation time constant. **F,** Activation (left panel) and deactivation time constant (right panel) in the absence (black circles) or the presence of either 66 or 200 μM AziPm (blue-shaded circles). AziPm only marginally increases the activation time constant but decreases the deactivation time constant.

```
  1   MEGGGKPNSS SNSRDDGNSV FPAKASATGA GPAAAEKRLG TPPGGGGAGA KEHGNSVCFK   60

 61   VDGGGGGGGG GGGGEEPAGG FEDAEGPRRQ YGFMQRQFTS MLQPGVNKFS LRMFGSQKAV  120
                                          HCNa               HCNb
121   EKEQERVKTA GFWIIHPYSD FRFYWDLIML IMMVGNLVII PVGITFFTEQ TTTPWIIFNV  180
         HCNc                         S1                  AziPm    AziPm
181   ASDTVFLLDL IMNFRTGTVN EDSSEIILDP KVIKMNYLKS WFVVDFISSI PVDYIFLIVE  240
       AziPm  S2                            S3a                   S3b
241   KGMDSEVYKT ARALRIVRFT KILSLLRLLR LSRLIRYIHQ WEEIFHMTYD LASAVVRIFN  300
                                S4
301   LIGMMLLLCH WDGCLQFLVP LLQDFPPDCW VSLNEMVNDS WGKQYSYALF KAMSHMLCIG  360
                S5                AziPm                  P-helix     SF
361   YGAQAPVSMS DLWITMLSMI VGATCYAMFV GHATALIQSL DSSRRQYQEK YKQVEQYMSF  420
                          S6                                A'-helix
421   HKLPADMRQK IHDYYEHRYQ GKIFDEENIL NELNDPLREE IVNFNCRKLV ATMPLFANAD  480
             AziPm  B'-hleix          C'-helix    D'-helix       E'-helix  F'-helix
481   PNFVTAMLSK LRFEVFQPGD YIIREGAVGK KMYFIQHGVA GVITKSSKEM KLTDGSYFGE  540
        A-helix                                                  AziPm
541   ICLLTKGRRT ASVRADTYCR LYSLSVDNFN EVLEEYPMMR RAFETVAIDR LDRIGKKNSI  600
       P-helix                     B-helix              C-helix
601   LLQKFQKDLN TGVFNNQENE ILKQIVKHDR EMVQAALPRE SSSVLNTDPD AEKPRFASNL  660
       D-helix                  E-helix
```

Fig. S2. Tandem mass spectrometry sequence coverage of digitonin-purified hHCN1-EM channels photolabeled with AziPm. 75% of the sequence (shown in black bold letters) was identified by tandem mass spectrometry. Photo-labeled sites are shown in red. Underlined sequences represent secondary structures and are labeled accordingly (blue). Cytosolic helices HCNa-c form the HCN domain. The transmembrane domain consists of the voltage sensing domain (VSD) and the pore domain. The VSD consists of S1 to S4 helices, followed by the pore domain comprised by helices S5 and S6. The cytosolic domain comprises the C-linker and the cyclic nucleotide-binding domain (CNBD). The C-linker is formed by A'- to F'-helices, and the CNBD consists of a region stretching from the A-helix to the E-helix. AziPm adducted residues are shown in red.

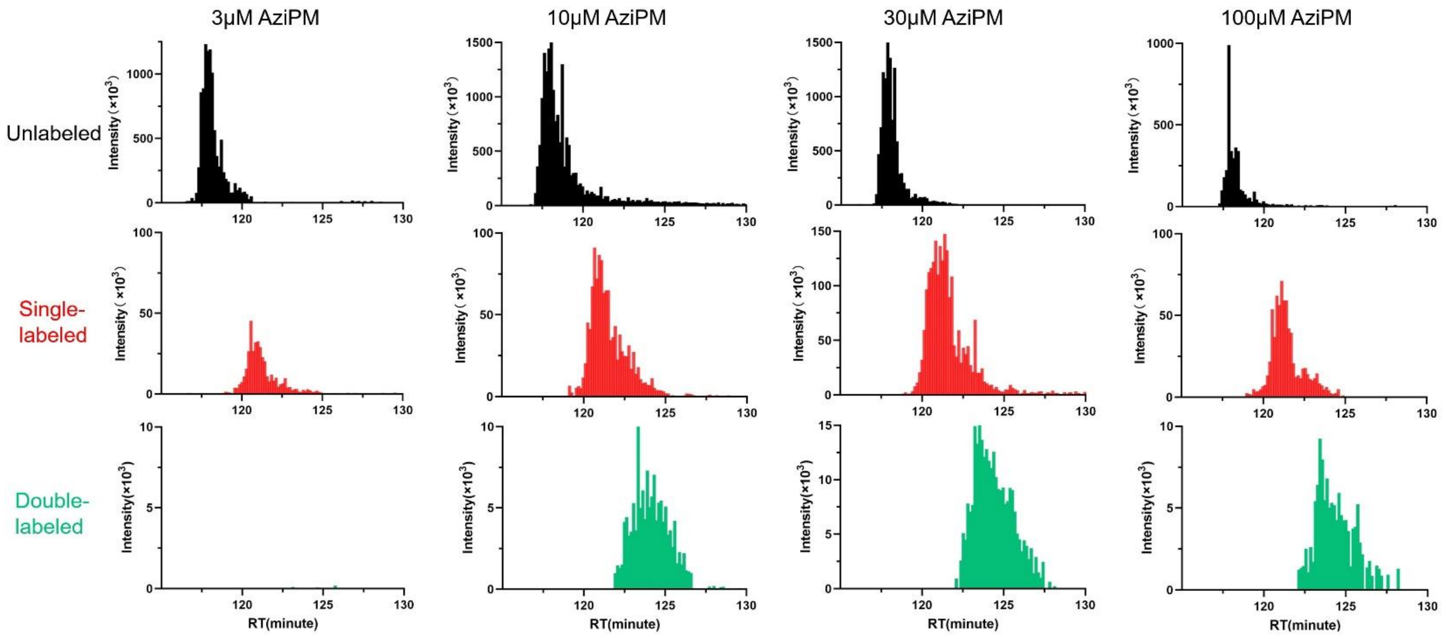

Fig. S3: Concentration-dependent photolabeling of the HCN1 S3-helix peptide by AziPm. Selected ion chromatograms showing the intensity of the unlabeled S3 peptide (top, black), the single AziPm-labeled peptide (middle, red) and the double AziPm-labeled peptide (bottom, green) as a function of AziPm concentration (3 – 100 μM). The chromatographic retention time of the peptides is increased from ≈119 min, to ≈122 min to ≈124 min by the successive addition of AziPm adducts. At 3 μM AziPm, only the single labeled peptide is observed indicating that the labeled Y234 residue is near the highest affinity binding site.

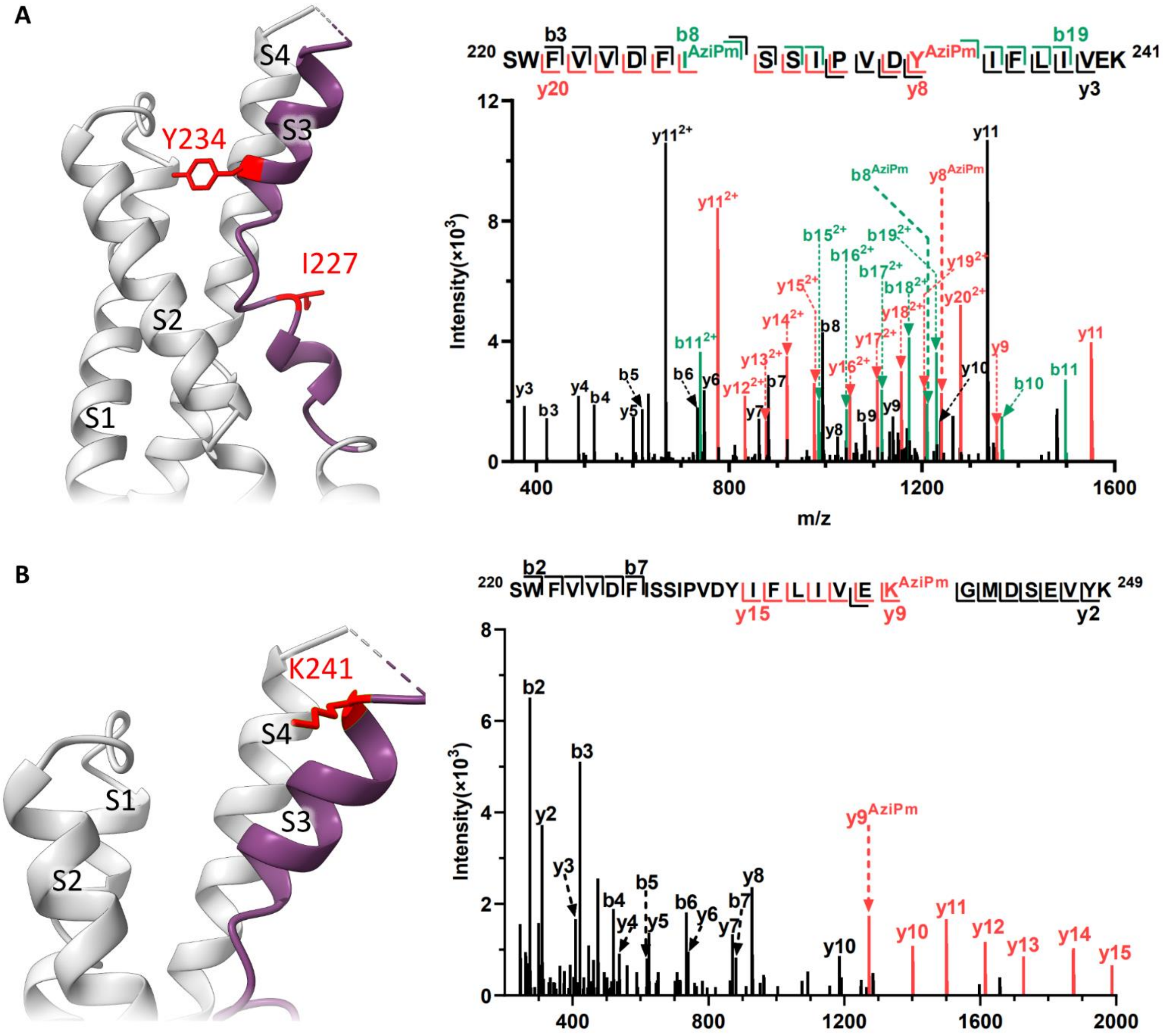

Fig. S4. Additional residues in the S3-helix peptide are labeled at high concentrations (100 µM) of AziPm. A, *Left panel*: Structural representation of the voltage sensor domain of HCN1 with the labeled S3 peptide highlighted in purple and adducted residues Y234 and I227 shown in red. *Right panel*: Fragment ion spectrum of a double-labeled S3 peptide indicating AziPm adduction at I227 and Y234. The labeled y8 ion and unlabeled y7 ion are site-defining for a Y234 adduct. The labeled b8 and unlabeled b7 ions are site-defining for an I227 adduct. **B,** *Left panel*: Structural representation of the voltage-sensor domain of HCN1 with a single-labeled S3 peptide with a missed tryptic cleavage highlighted in purple and the adducted K241 residue shown in red. *Right panel*: Fragment ion spectrum of an S3 peptide labeled at residue K241. The labeled y9 and unlabeled y8 fragment ions are site-defining for AziPm adduction at K241. In both fragment ion spectra (a and b), y-ions containing the AziPm adduct are shown in red and b-ions containing the adduct are shown in green. Fragment ions not containing the adduct are shown in black.

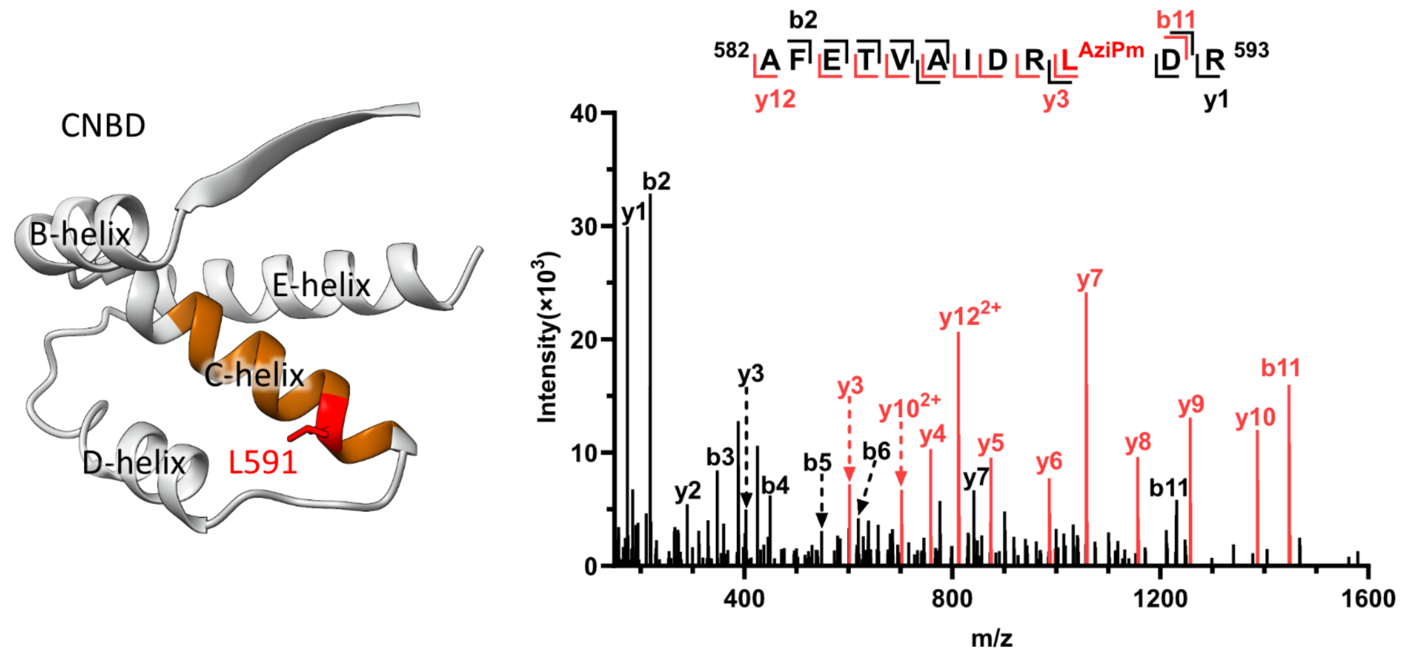

Fig. S5. AziPm labels the C-helix of the CNBD. *Left panel*: Structural representation of the cyclic nucleotide binding domain (CNBD) of HCN1 with the labeled C-helix peptide highlighted in brown and the likely adducted L591 residue shown in red. *Right panel*: Fragment ion spectrum of a C-helix peptide labeled with AziPm. The labeled y3 and unlabeled y4 fragment ions coupled with the labeled b11 fragment ion identify L591 as the adducted residue.

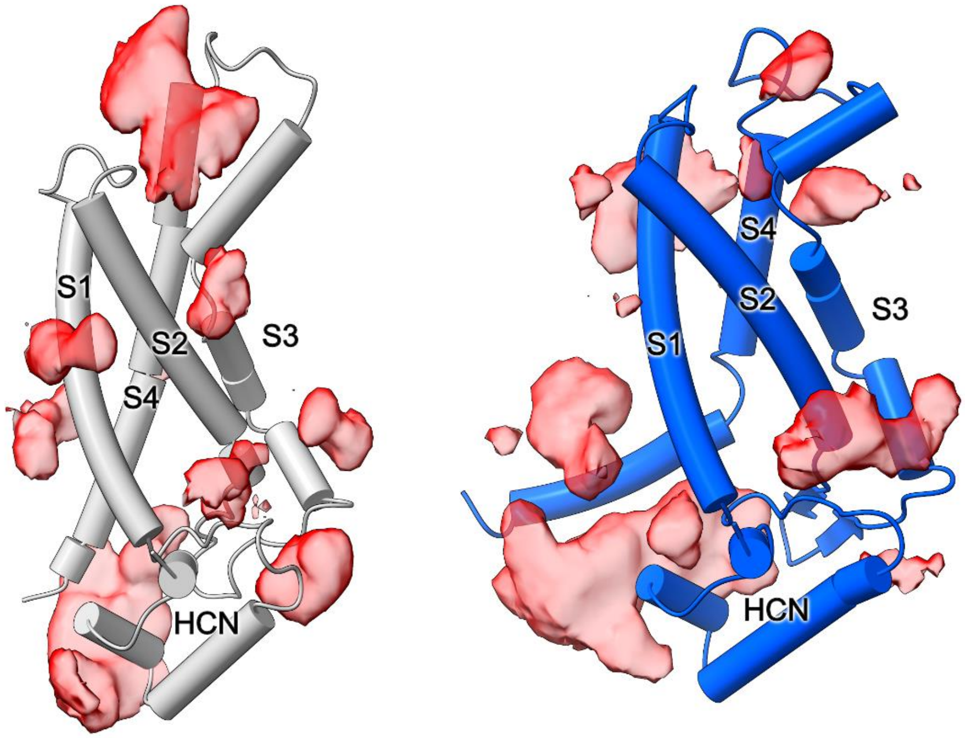

Fig. S6. Propofol Density Maps Derived from Atomic MD Simulations. Time-averaged propofol density maps obtained from the closed state (PDB: 5U60, left panel) and the activated voltage sensor domain state (PDB: 6UQF, right panel) are shown in red (contour level: 0.015). These maps illustrate the spatial distribution of propofol binding sites under different conformational states of the protein. The density maps were generated using two sets of 500 ns molecular dynamics simulation, pre-equilibrated with 100 mM propofol.

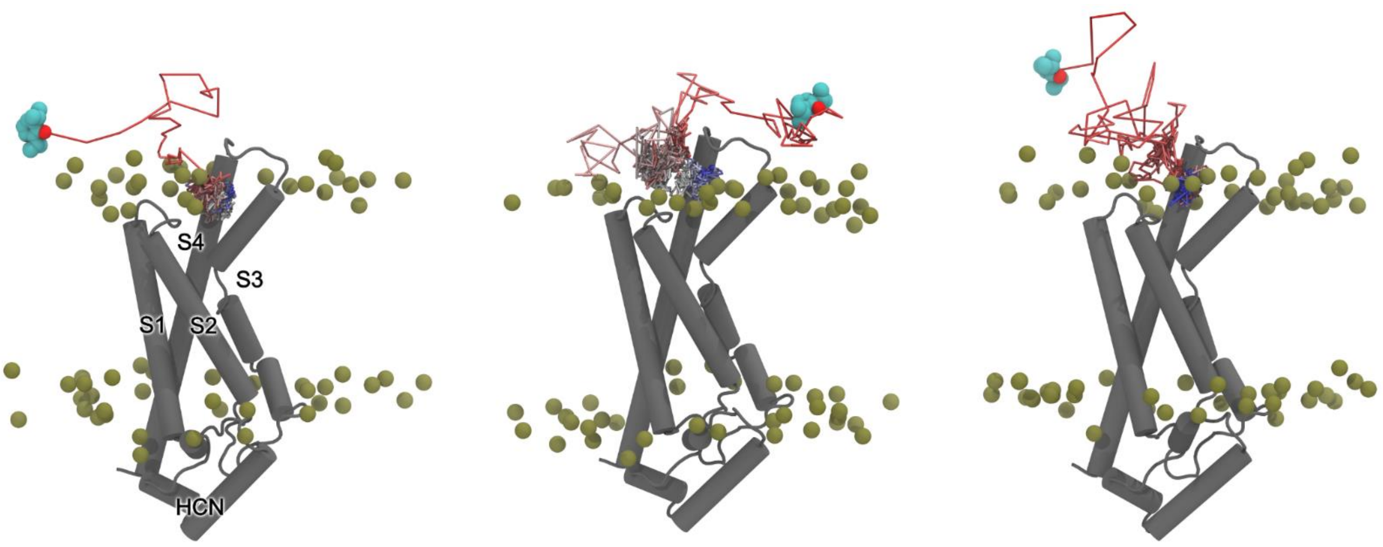

Fig. S7. Trajectories of Three Independent Propofol Binding Events in the voltage-sensing domain of the Closed state structure of HCN1. Propofol (cyan) molecules at the start of the simulations are depicted in space-filling representations. The time trajectories of various propofol molecules are color-coded, with the initial position in red and the final position in blue. The protein structure is represented as grey tubes, and the phospholipid head groups are shown as olive spheres.

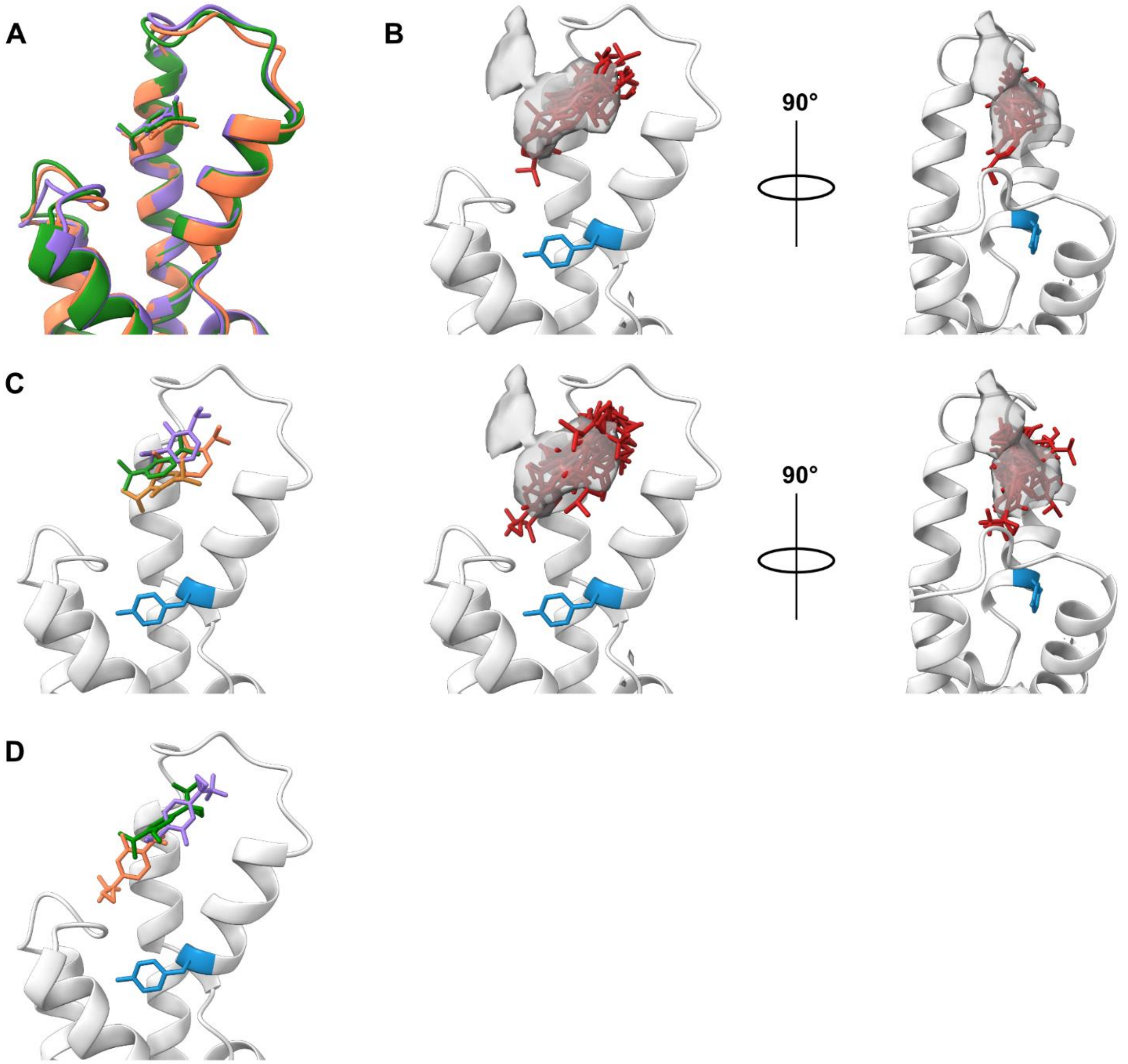

Fig. S8. Comparison of propofol binding poses from atomistic MD-simulations and molecular docking. A, Top binding poses in atomistic flooding simulations from PyLipID analysis. Top pose is shown in salmon, second ranked pose shown in purple, and third ranked pose shown in green. **B,** Top five docking poses to each of the three conformations in A for propofol (top) and AziPm (bottom). Docking poses are overlayed with the probability density for propofol in flooding simulations shown at 0.035 threshold. The photolabeled Y234 is shown in blue for reference. **C,** Top docking poses (salmon, purple, and green) overlayed with the top ranked pose from PyLipID (brown). **D,** Top ranked docking poses for AziPm colored according to the scheme in panel A.

Table S1. Comparison of the potential of half-maximal channel activation in the absence and the presence of various propofol concentrations between mHCN1-WT and mutant channels.

mHCN1 WT and mutants	Residue position hHCN1	N	Control	Propofol (µM) 0.3	1	3	10	30	100
			$V_{0.5}$ (mV)						
**WT**		9	-66.4 ± 2.9	-68.9 ± 2.0	-73.5 ± 2.2	-81.3 ± 2.1	-91.0 ± 1.5	-101.0 ± 1.4	-110.5 ± 1.4
**Q159W**	**Q170**	5	-125.1 ± 2.1	nd	nd	nd	-130.0 ± 2.0	nd	-137.9 ± 2.3
**L226A**	**L237**	8	-102.1 ± 1.0	nd	nd	nd	-115.9 ± 1.0	nd	-124.9 ± 2.6
**E229A**	**E230**	9	-87.9 ± 1.5	nd	nd	nd	-95.2 ± 1.1	nd	-85.9 ± 1.9
**K230A**	**K241**	9	-89.7 ± 2.4	nd	nd	nd	-109.1 ± 1.4	nd	-136.0 ± 2.7
**A242W**	**A253**	1	-68.7 ± 1.5	nd	nd	nd	-109.1 ± 1.4	nd	-136.0 ± 2.7

Data are given as mean ± SE. nd: no data

Table S2. List of mutagenesis primers

mHCN1 WT and mutants	Residue position hHCN1	Forward primer (5' to 3')	Reverse primer (5' to 3')
**Q159W**	**Q170**	TCACAGAG**tg**GACGACAACACCG TGGATTATTTTC	TTGTCGTC**ca**CTCTGTGAAGAAC GTGATTCCAACT
**L226A**	**L237**	TATCTTT**gcc**ATTGTAGAGAAAGG GATGGACTCAGA	CTCTACAATggcAAAGATATAATC CACCGGGATCGATGAG
**E229A**	**E230**	CATTGTAG**cc**AAAGGGATGGACTC AGAAGTTTACAAG	ATCCCTTT**gg**CTACAATGAGAAAG ATATAATCCACCGGG
**K230A**	**K241**	TTGTAGAG**gc**AGGGATGGACTCA GAAGTTTACAAG	CCATCCCT**gc**CTCTACAATGAGAA AGATATAATCCACCGG
**A242W**	**A253**	CAGCCAGA**tgg**CTTCGTATCGTGA GGTTTACAAAAATTC	TACGAAG**cca**TCTGGCTGTCTTGT AAACTTCTG

Bold lowercase letters indicate the substituted nucleotides.
